# Oculometric biomarkers of visuomotor deficits in clinically asymptomatic patients with systemic lupus erythematosus undergoing long-term hydroxychloroquine treatment

**DOI:** 10.3389/fopht.2024.1354892

**Published:** 2024-07-22

**Authors:** Andrew R. Berneshawi, Kimia Seyedmadani, Rahul Goel, Mark R. Anderson, Terence L. Tyson, Yasmin M. Akay, Metin Akay, Loh-Shan B. Leung, Leland S. Stone

**Affiliations:** ^1^ Ophthalmology Department, Stanford University School of Medicine, Stanford, CA, United States; ^2^ Research Operations and Integration Laboratory, Johnson Space Center, National Aeronautics and Space Administration, Houston, TX, United States; ^3^ Biomedical Engineering Department, University of Houston, Houston, TX, United States; ^4^ San Jose State University Foundation, San Jose, CA, United States; ^5^ Human Systems Integration Division, Ames Research Center, National Aeronautics and Space Administration, Moffett Field, CA, United States; ^6^ Arctic Slope Regional Corporation (ASRC) Federal Data Solutions, Moffett Field, CA, United States

**Keywords:** pursuit eye movements, saccadic eye movements, visual motion processing, retinal function, surveillance, structure-function analysis

## Abstract

**Introduction:**

This study examines a set of oculomotor measurements, or “oculometric” biomarkers, as potential early indicators of visual and visuomotor deficits due to retinal toxicity in asymptomatic Systemic Lupus Erythematosus (SLE) patients on long-term hydroxychloroquine (HCQ) treatment. The aim is to identify subclinical functional impairments that are otherwise undetectable by standard clinical tests and to link them to structural retinal changes.

**Methods:**

We measured oculomotor responses in a cohort of SLE patients on chronic HCQ therapy using a previously established behavioral task and analysis technique. We also examined the relationship between oculometrics, OCT measures of retinal thickness, and standard clinical perimetry measures of visual function in our patient group using Bivariate Pearson Correlation and a Linear Mixed-Effects Model (LMM).

**Results:**

Significant visual and visuomotor deficits were found in 12 asymptomatic SLE patients on long-term HCQ therapy compared to a cohort of 17 age-matched healthy controls. Notably, six oculometrics were significantly different. The median initial pursuit acceleration was 22%, steady-state pursuit gain 16%, proportion smooth 7%, and target speed responsiveness 31% lower, while catch-up saccade amplitude was 46% and fixation error 46% larger. Excluding the two patients with diagnosed mild toxicity, four oculometrics, all but fixation error and proportion smooth, remained significantly impaired compared to controls. Across our population of 12 patients (24 retinae), we found that pursuit latency, initial acceleration, steady-state gain, and fixation error were linearly related to retinal thickness even when age was accounted for, while standard measures of clinical function (Mean Deviation and Pattern Standard Deviation) were not.

**Discussion:**

Our data show that specific oculometrics are sensitive early biomarkers of functional deficits in SLE patients on HCQ that could be harnessed to assist in the early detection of HCQ-induced retinal toxicity and other visual pathologies, potentially providing early diagnostic value beyond standard visual field and OCT evaluations.

## Introduction

Hydroxychloroquine (HCQ) is a widely prescribed medication for the treatment of autoimmune diseases, such as rheumatoid arthritis and *Systemic Lupus Erythematosus* (SLE). However, its prolonged use carries a risk of retinal toxicity, potentially leading to irreversible visual impairment (1–4). Given the importance of early detection in preventing further visual deterioration, there is a growing interest in identifying potential biomarkers for the early diagnosis of HCQ retinal toxicity.

A critical diagnostic tool in the surveillance of HCQ toxicity is Spectral Domain Optical Coherence Tomography (SD-OCT) to monitor for specific patterns of retinal thinning. However, this approach can only detect disease progression once retinal thinning has become evident. Furthermore, another challenge is determining at what point structural changes result in clinically detectable functional impairment. Therefore, there is a clear clinical need for a behavior-based method to track disease progression. Such an approach could potentially enable earlier detection and intervention with potentially better preservation of visual function.

Recently, there has been renewed interest in using oculography as a potential clinical tool for aiding in the diagnosis of various ophthalmic and neurological conditions (5–10). The goal of this study is to evaluate the potential value of a specific set of biomarkers derived from voluntary ocular tracking responses (11, 12) as a tool for monitoring retinal health in patients undergoing chronic HCQ treatment, and possibly other populations at risk of slowly progressive retinal disease (13–16).

Eye movements are essential for humans to explore, gather, and process visual information from the environment. Specifically, they enable humans to point their fovea, the region of the retina with the highest visual acuity, at visual features or objects of interests and, when necessary, to track them as they move. Maintaining foveation on attended objects of interest in a dynamic world supports general sensorimotor coordination, visual processing, and ultimately a better cognitive understanding of the scene (17–21). During steady-state tracking, the seamless coordination of smooth pursuit and corrective “catch-up” saccades (22) enables foveal (or at least near foveal) targeting and stabilization by responding to a host of factors, including residual retinal position and velocity errors (23–26), visual perception (27–32), as well as attention, anticipation, prediction, and other cognitive factors (33–42).

The details of the execution of eye movements thus contain a wealth of information about neural processing that has long been known to have diagnostic clinical relevance (7, 43–52) and has more recently been shown to capture more subtle, subclinical decrements in neural function (11, 12, 53–55). In particular, we have used the combined smooth and saccadic eye movements used to track moving objects to compute a set of “oculometric” measures of human visual and visuomotor function (56, 57), each largely independent from the other (11). We have previously demonstrated that a 5-to-10-minute ocular tracking task is sufficient to measure these oculometric variables reliably (58) and to detect and characterize mild impairment due to mild-to-moderate traumatic brain injury, low-dose alcohol consumption, and sleep deprivation (7, 11, 12, 55).

The goal of this study is two-fold. First, in an across-subject study, we aim to investigate whether oculometric measures of visuomotor performance can detect and characterize potential mild impairment in a cohort of visually and neurally asymptomatic patients undergoing chronic HCQ treatment relative to a cohort of age-matched healthy controls. Second, in a within-subject study, we aim to determine the relationship between structural and functional variation across this at-risk population by examining the relationship between oculometric measures and retinal thickness. We hope our findings will contribute to the development of novel diagnostic approaches for the early detection and assessment of retinal damage in patients under prolonged HCQ treatment by identifying and validating correlations between experimental functional eye-movement metrics and established structural retinal measures known to capture clinical pathology. Additionally, our findings may provide insights into the relationship between oculomotor responses and retinal thickness in general, thus broadening our understanding of the value of eye movements as a rapid, non-invasive method of assessing retinal health.

## Materials and methods

### Ethical approval

The study conformed to the standards set by the latest revision of the Declaration of Helsinki, except for registration in a database, with each participant providing their informed, written consent prior to their participation. The Stanford portion of the human research data collection was conducted at the Byers Eye Institute, Stanford University School of Medicine, and was approved by the Institutional Review Board of Stanford University. The National Aeronautics and Space Administration (NASA) portion of the human research data collection was conducted at Johnson Space Center (JSC) and was approved by the NASA Human Research Institutional Review Board.

### Participants

We recruited two distinct cohorts for this study. The patient group consisted of 12 individuals with SLE undergoing HCQ treatment at the Byers Eye Institute. These participants were confirmed to be otherwise in overall good health by their eye care provider. The control group comprised 17 participants invited from the Johnson Space Center (JSC) clinic subject pool. These participants underwent health pre-screening at the JSC clinic, including for normal visual acuity, color vision, depth perception, visual field, and intraocular pressure measures.

For both groups, proficiency in English was required. All participants were instructed to maintain regular sleep habits, defined as attempting to get 8.5 hours of sleep the night prior to testing (the patient group self-reported getting an average of 7.8 hours of sleep, while the control group reported an average of 7.7 hours), and to abstain from caffeine, alcohol, nicotine, and recreational drugs for at least 24 hours prior to the test. We excluded participants with a history of Traumatic Brain Injury (TBI), insomnia or sleep disorders, recurring neck or back pain, neurological pathologies or injuries, serious hearing impairments, visual acuity not correctable to 20/40 or better, or who had consumed alcohol, marijuana, tobacco or recreational drugs in the 24 hours before the study. Additionally, individuals using certain medications, including opioids, benzodiazepines, or other neuroactive agents associated with neurologic impairment, were also excluded. All participants self-reported being free of neurological illnesses or conditions, with no hospitalization or loss of consciousness due to head injury.

### Eye tracking and stimulus presentation

All participants (patients and controls) performed the identical oculomotor task in matched set-ups at JSC and at Byers Eye Institute, under the same conditions, using identical displays, eye-trackers, and data-collection and analysis software. We utilized a video-based, table-mounted pupil-tracking system, with an accuracy of approximately 0.5 degrees and a precision of about 0.2 degrees, in conjunction with an HD-resolution 144 Hz BenQ model XL2420Z display (59).

The oculomotor task used in this study was an adapted radial version of the Rashbass step-ramp paradigm (56, 60). Our paradigm allows for a randomized sampling of polar angles of target motion over the course of 90 trials, with trials occurring every 4 radial degrees around the clock dial (12). A session began by adjusting the participant’s seating height position with respect to the chin and forehead rest for optimal ergonomic comfort. Viewing distance was 46 cm from the display screen. Next, we calibrated the system for each participant by having them fixate targets in nine locations within a 3 x 3 Cartesian grid subtending ±10 deg horizontally and vertically (29), then at two eccentric locations (± 30 deg) to assess gaze holding, and finally at central gaze with the background flashing on and off to assess the pupillary light response. After the calibration routine, a 0.2-degree fixation spot was presented in the middle of the screen at the beginning of each trial. Participants initiated the trial by fixating on the fixation spot and pressing a button on a game controller when they were ready to track (self-paced task). The target would then jump 2.5–3.8 deg away from the central fixation location in a random direction after a random duration of time between 200 to 5,000 ms. Immediately afterwards, the target moved in the opposite direction, back towards the center of the screen at a constant speed (16, 18, 20, 22, or 24 deg/s) for a random period of time between 700 and 1,000 ms before disappearing. Participants were instructed to maintain their focus on the target without blinking and to follow it with their eyes as accurately as possible until it vanished. The fixation spot would then reappear at the center, prompting the participant to initiate the next trial in the self-paced task.

### Oculometric analysis

Eye-movement data were preprocessed to remove artifacts and blinks and to detect saccades as outlined previously (61). Nineteen largely independent oculometrics were measured using established analyses (described in detail in 11, 12) and computed in MATLAB™ (R2023a, The MathWorks, Natick, MA, USA):

- “Latency” is the duration between the onset of the target moving and the beginning of the participant’s smooth eye-movement (pursuit) response to follow the target. This metric captures the initial response time of the pursuit system (24, 25).
- Initial “Acceleration” is the rate at which the eye-movement speed initially increases in the first 100-ms from the onset of the tracking response. This metric captures the robustness of the initial (open-loop) pursuit response (25).
- Steady-state “Gain” is the mean eye speed during the saccade-free component of the steady-state tracking response (400–700 ms after target motion onset), projected along the target direction and divided by the target speed. This metric captures the robustness of the steady-state (closed-loop) pursuit response (24).
- “Proportion Smooth” is the proportion of time that the steady-state tracking response is smooth pursuit as opposed to a saccade.
- “Saccadic Rate” is the total number of catch-up saccades occurring in the steady-state tracking interval divided by the total steady-state tracking time (300 ms per trial plus any added lead time if a saccade onset preceded and the saccade spanned the initial interval boundary). Trials with blinks in the steady state intervals were excluded from this analysis, but this rarely occurred.
- “Saccadic Amplitude” is the amplitude of the forward catch-up saccades occurring during the steady-state tracking interval, projected onto the axis of target motion. This metric captures the magnitude of the offset correction achieved by each catch-up saccade measured.
- “Saccadic Dispersion” is the standard deviation of the distribution of directions across the distribution of forward catch-up saccades. This metric is a measure of dynamic spatial localization and captures the directional variability in saccade generation convolved with any initial pursuit trajectory error. Thus, it is more difficult to anticipate whether it should increase or decrease with impairment.
- “Direction Noise” is the standard deviation of eye-movement direction during the initial 160-ms of pursuit. This metric captures the precision of the visual direction signal driving the pursuit response (56) and is related to the uncertainty in direction perception (32).
- “Direction Anisotropy” is the 2^nd^ harmonic of the slope of the change of pursuit direction as a function of target direction. This metric is a measure of direction (in)accuracy and captures the well-known oblique effect whereby direction discrimination varies as a four-fold cloverleaf around the clock with increased performance near the cardinal directions and decreased performance near the obliques (56).
- “Direction Asymmetry” is a second measure of direction (in)accuracy and captures the systematic, but idiosyncratic, horizontal-vertical bias (1^st^ harmonic) of the direction tuning curve.
- “Speed Noise” quantifies the standard deviation of steady-state pursuit eye speed divided by mean eye speed. This metric captures the precision in the underlying speed signal driving steady-state pursuit and is related to the Weber fraction for speed perception (28).
- “Speed Responsiveness” is the slope of the change in median eye speed (for responsive trials above a 4 deg/s threshold) as a function of small changes in target speed. This metric captures the accuracy of speed signals driving steady-state pursuit.
- The “Main Sequence Slope” and “Main Sequence Intercept” are the slope and intercept, respectively, of the peak velocity versus amplitude curve for saccades (62). These two metrics capture the health of the brainstem saccadic generator.
- “Tau C” and “Tau D” are the dominant time constants of the pupillary contraction and dilation responses to light onset and offset, respectively. These two metrics capture the health of subcortical non-image-forming visual pathways.
- “Lateral Drift” and “Centripetal Drift” are slow smooth drift movements of the eyes measured with respect to the world and to central gaze, respectively, when fixating an eccentric target at ±30 deg during the calibration procedure. Such eye movements could indicate a latent directional or gaze-evoked nystagmus due to cortical, cerebellar, or peripheral vestibular pathology.

For this paper, we added an additional oculometric measure, “Fixation Error”, to capture the difficulty that some patients had reliably fixating during the calibration. Fixation Error is the mean fixation absolute unsigned error across the 9-point calibration grid recorded during the calibration routine. This metric captures the static-localization performance of the fixation system, and should not be confused with the calibration accuracy, which is the mean signed random residual fixation error, which was similarly small in both groups (for patients, the mean was 0.21 deg; for controls, 0.19 deg).

### Clinical measures

Retinal “Thickness” is the average thickness of the nine macular sectors, as defined by the Early Treatment Diabetic Retinopathy Study, measured from the internal limiting membrane to the retinal pigmented epithelium using spectral-domain OCT (CIRRUS HD-OCT 5000 OCT, Zeiss^TM^, Dublin, CA). Normative data for this measure have been published previously (63).

The “Mean Deviation (MD)” is a global index used in Humphreys 10–2 visual-field assessment that represents the average difference in sensitivity in the visual field compared to normal values. A negative MD value indicates overall depression of light sensitivity in the visual field.

The “Pattern Standard Deviation (PSD)” from the Humphreys 10–2 visual-field test represents the irregularity or the degree of localized visual field defects. PSD is calculated by comparing the patient’s visual field test results to a normative database. The square root of the average squared deviation from baseline gives the PSD, which is a measure of the pattern of visual field loss. A higher PSD value indicates more localized defects and less uniformity in the visual field.

The “Age” of subjects is their age in years on their last birthday before data collection. In the rest of the paper, we capitalize all references to the specific ocular, clinical, and demographic metrics when used as defined in quotes above in the Methods (e.g., Latency, Acceleration, Gain, Thickness, Age, etc.) and will use the same terms uncapitalized when referring to their more generic meanings.

### Statistical analysis

To evaluate the difference between oculometrics across the two experimental groups, we performed Mann-Whitney *U* tests for all metrics except for the two drift metrics for which we used a one-sample Wilcoxon Signed-Rank test of significance with respect to a theoretical zero healthy baseline. We used one-tailed tests for most metrics, justified by our prior hypothesis of impairment in the patient group given an objective sign associated with impairment (e.g., Latency longer, Acceleration lower, Gain lower, etc.). For six metrics (Saccadic Dispersion, Direction Anisotropy, Direction Asymmetry, Main Sequence Slope, Main Sequence Intercept, Lateral Drift), for which there is no clear *a priori* expected direction for the impairment, we used a two-tailed criterion.

To evaluate the relationship between visuomotor function and Thickness or other clinical measures, we first performed standard bivariate Pearson correlation analyses between the oculomotor metrics and Thickness across retinae (raw or normalized, i.e., converted to z-scores based on Zeiss’ reported age- and sex-corrected percentiles). We used a t-test to compute the significance of the correlation while conservatively setting the degrees of freedom to the number of subjects minus two (i.e., df = 10 for all *r* ^2^ reported below, except for the correlations with MD for which df = 8 because there were two patients without MD measurements) to avoid any artifact due to correlation between the two retinae by assuming the worst case. To account for any potential effect of Age as well as Thickness and to better handle the repeated measures across the two retinae tested for each subject, we also used a Linear Mixed-effects Model (using a co-variance structure of compound symmetry) to determine whether either of these effects was significant using a proper estimate of the degrees of freedom (reported for each test below) and to determine the maximum likelihood estimate of Thickness and Age fixed effects with no interaction term or random effects in the model. For the correlation and LMM analyses, we again used a one-tailed criterion for those metrics for which there is a clear prior hypothesis as to the sign of impairments and correlation, and two-tailed tests otherwise (see above). Lastly, because the above statistical analyses for the various oculometrics are testing individually distinct hypotheses (i.e., is this particular metric, and the specific function it captures, impaired compared to the control group, or correlated with a particular clinical measure?), we did not correct for multiple tests when reporting *p* values, consistent with previous studies (11, 12).

## Results

### Demographics and clinical assessment

To detect an impairment in visual or visuomotor function (if any) in our patient group, in the absence of pre-SLE/HCQ data, we compared performance between two groups of subjects in an across-subject experimental design (**Table 1**). Our group of patients had been receiving long-term HCQ treatment (mean treatment time: 12.7 years, range: 2 – 26 years) for their diagnosed SLE. They were screened to be free of neurological pathologies and well rested, with no current recreational drug use, at least 20/40 corrected acuity in both eyes, and no alcohol or caffeine consumption in the 24 hours prior to testing. In particular, visual acuity (VA) was measured as part of their routine clinical testing; their median VA had a logMAR of 0 (range: 0 to 0.18) with a mean of 0.03 and 19 of 24 eyes having a corrected acuity of 20:20. Thus, their VA was typical of the general population. Furthermore, we have previously shown that there is no significant correlation between oculometric measures and acuity in this range (57), so any small residual systematic difference in acuity between our patient and control groups would not be expected to affect our results. Lastly, none in our patient group reported visual symptoms and were thus all deemed clinically asymptomatic, although two showed definitive clinical signs of early HCQ toxicity based on parafoveal thinning in their OCT data. Our group of age-matched healthy controls was similarly screened and used to represent normal baseline human performance.

**Table 1:** Parameters of our patient and control groups.

		Demographic Measures		Clinical Measures		
Table 1 Subjects	*N*	Sex	Median Age [range] (yrs)	Median Thickness [range] (μm)	Median Mean Deviation [range]	Median Pattern Standard Deviation [range]
Patients	12	12F	34 [26 - 62]	274.5 [194 - 299]	0.10 [-6.59 - 2.41]	1.21 [0.92 - 3.74]
Controls	17	8F/9M	35 [24 - 55]	277.5 [250 - 312]†	> -2‡	< 2‡

Only two of the 12 patients were diagnosed with mild toxicity from a qualitative and quantitative clinical examination of their OCT data. Two patients showed OCT average thickness below 250 µm (one of them was one of the two with diagnosed toxicity; the other was not but had thickness measurements of 246 and 245 in the two eyes, respectively, slightly outside the normal range). Two different patients showed MD < -2 in at least one eye. Despite the potential deviations from strictly normal in 5 of the 12 Patients, all were asymptomatic as they did not report any visual symptoms. †We did not have access to OCT data from our control group, so the baseline Thickness values in **Table 1** are from a large population (N = 112) database published elsewhere (63). ‡The baseline MD/PSD values are also not from our control group, but represent typical threshold values for healthy used in current clinical practice (64). However, the arbitrary thresholds shown for normal MD and PSD are not universally accepted and were chosen to be conservative. Note that the median values of Thickness, Mean Deviation (MD), and Pattern Standard Deviation (PSD) of our patient group are all well within the normal range.

Note that our patient group had, on average, normal macular thickness as compared to published baseline values for a mixed-sex population (63), although two of our patients has at least one eye with a value below the reported lowest value of the normal range. Note that although this study (63) found that the median macular thickness value for females is slightly lower than for males (276.6 vs 280.7 µm), this small difference does not alter the above statement. Our patient group had, on average, normal MD and PSD, although two subjects had MD values below that conservatively deemed normal (-2). The correlation of MD with Treatment Duration nearly reached significance (*r* ^2 ^= 0.223, *p* = 0.084), but with PSD was not close (*r* ^2 ^= 0.0003, *p* = 0.480). The correlation between Thickness and Treatment Duration (*r* ^2 ^= 0.106, *p* = 0.151) and between Thickness and Dosage (*r* ^2 ^= 0.061, *p* = 0.220) were not significant.

**Figure 1:**
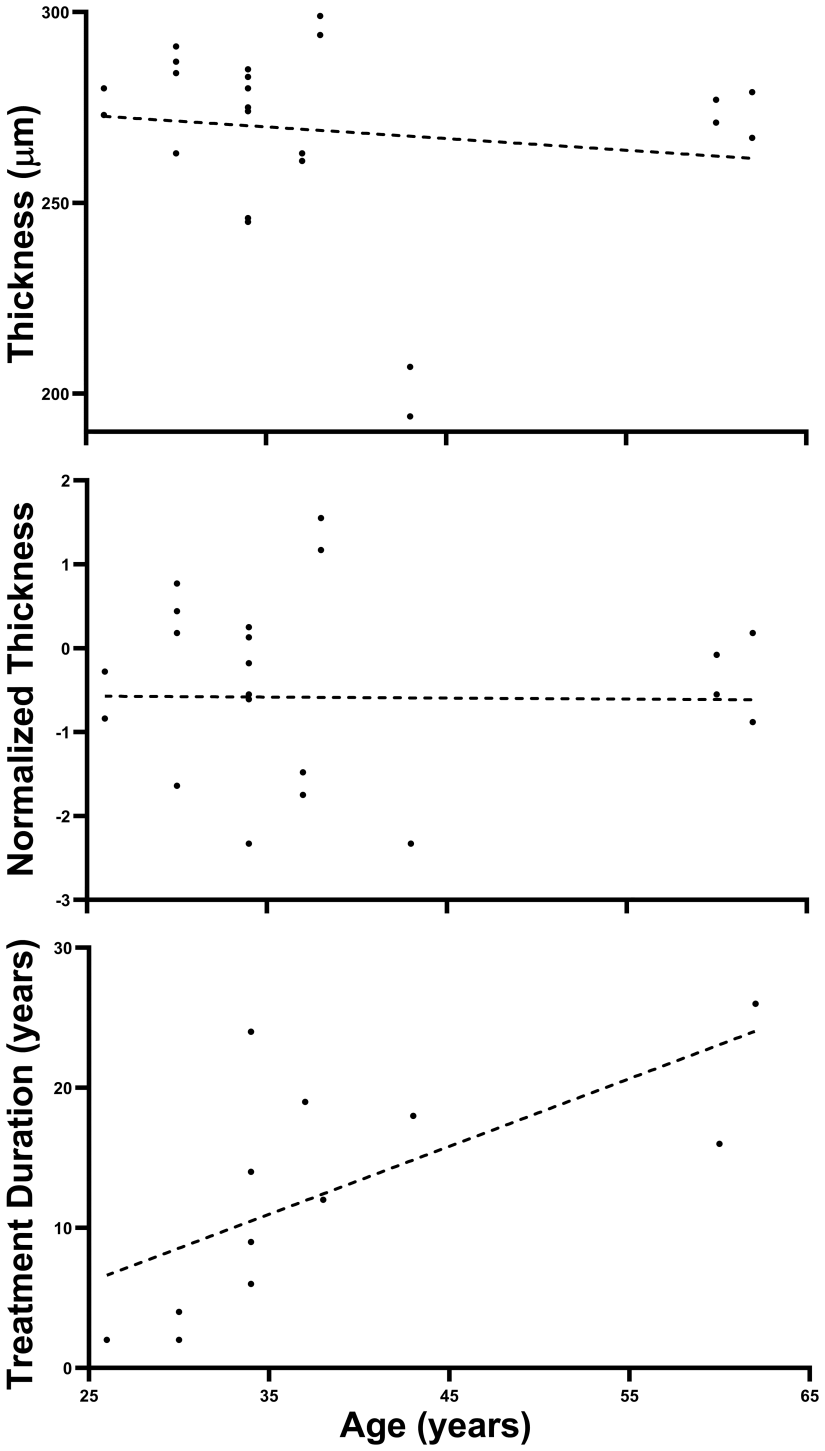
Absence of Age effect on retinal thickness. The three panels above plot Thickness, Normalized Thickness, and HCQ Treatment Duration as a function of Age for our Patient population (all plots have 24 points representing 24 retinae although some superimpose). Note that only HCQ Treatment Duration shows a significant linear trend (as expected). Dashed lines are best fitting linear-regression lines.

**Figure 1** shows that, although Treatment Duration was highly correlated with Age as the older patients in our patient group have required treatment for longer periods of time (*r* ^2 ^= 0.444, *p* = 0.009), Thickness was not correlated with Age (*r* ^2 ^= 0.019, *p* = 0.336) in our patient group, consistent with previous findings in a population of 112 healthy controls (63). Furthermore, Normalized Thickness in our patients is by design devoid of any hint of correlation with Age (*r* ^2 ^= 0.00013, *p* = 0.486).

### Functional impairment in our patient group

In contrast with their generally normal clinical measures of retinal function (**Table 1**), our patient group exhibited notable impairments in certain aspects of visuomotor function (first row of **Table 2**) when compared to our control group (third row of **Table 2**). In particular, the median values demonstrated slower Acceleration (22%), reduced Gain (16%), lower Speed Responsiveness (31%), and an increase in catch-up Saccade Amplitude (46%) associated with a lower Proportion Smooth (7%) when tracking moving targets. They also showed significantly increased Fixation Error (46%) when viewing stationary targets. There was also a suggestion of an elevation in Saccadic Dispersion that did not quite reach significance. Excluding the two in our patient group who showed mild toxicity in their OCT data, we found that four metrics continued to show significant deficits, despite the absence of toxicity (second row of **Table 2**).

**Table 2:** Median oculometric responses.

Table 2 Functional Effects	Early Perifoveal Vision		Late Foveal Vision	Visual Motion Precision		Visual Motion Accuracy			Subcortical Visuomotor Response				Pupillary Response		Localization
	Latency (ms)	Acceleration (deg/s^2^)	Gain	Direction Noise (°)	Speed Noise (%)	Direction Anisotropy	Direction Asymmetry	Speed Responsiveness	Saccadic Rate (Hz)	Saccadic Amplitude (deg)	Saccadic Dispersion (°)	Proportion Smooth	Contraction Time Constant (ms)	Dilation Time Constant (ms)	Mean Fixation Error (deg)
Patient Median (N = 12)	165.5	90.5	0.76	9.4	17.5	0.30	0.01	0.36	3.53	2.19	*21.4*	0.71	172	911	0.73
No-tox Patient Median (N = 10)	161.5	90.5	0.79	9.1	16.8	0.30	-0.09	**0.36**	3.29	2.11	*21.4*	0.70	172	911	0.68
Control Median (N = 17)	164.0	116.0	0.90	9.1	15.4	0.24	0.05	0.52	3.51	1.50	15.7	0.76	176	756	0.50
p (Mann-Whitney U test)	0.4870 0.2722	0.0003 0.0003	0.0101 0.0321	0.5000 0.3696	0.1516 0.3196	0.8532 0.2914	0.6873 0.1462	0.0207 0.0451	0.3394 0.4510	0.0002 0.0013	0.0949 0.0504	0.0371 0.0870	0.4008 0.2931	0.3594 0.3217	0.0442 0.1022

Note that six metrics show significant (p < 0.05) differences between the patient and control groups and that four metrics remain significantly different even when the patient group is limited to the ten without diagnosed retinal toxicity. Significant impairments (p < 0.05) are bolded and those that approach significance (p < 0.11) are italicized. Top p-values are for the comparison of controls with the full patient population of twelve, while the bottom values are for comparison with the population of ten without HCQ toxicity.

We found no evidence of impairment in Latency, Direction Anisotropy, Direction Asymmetry, Direction Noise, Speed Noise, or in the pupillary light response. Furthermore, several of our oculometrics that capture primarily the performance of the efferent limb of oculomotor system showed little or no evidence of impairment. Specifically, we found no evidence of Lateral or Centripetal Drift (median of both < 0.005 deg/s, *p* = 0.791 and *p* = 0.485, respectively) suggesting healthy optokinetic and cerebellar pathways, and only a hint of mildly altered brainstem saccadic generation (p = 0.059 and *p* = 0.123 for the slope and intercept of saccadic velocity vs. amplitude curve, respectively) that may just be an artifact of the significantly larger saccadic amplitudes in the patient group.

In summary, significant impairment of our patient group relative to our control group was observed for 6 of the 19 tested oculometrics with an additional oculometric measure almost reaching significance.

### Correlations between structural and functional metrics of retinal health

To examine the relationship (if any) between the observed oculometric deficits (**Table 2**) and retinal health, we performed a within-subject correlation analysis between our oculometric measures and clinical measures of retinal structure (OCT) and function (perimetry) in our patient population. **Table 3** shows the correlation analysis results for those oculometric measures that exhibited significant or nearly significant correlations with Thickness or MD. Standard clinical measures of visual function (MD and PSD) were not correlated with Thickness (*r* ^2 ^= 0.003, *p* = 0.437 and *r* ^2 ^= 0.026, *p* = 0.330, respectively), and this remained unchanged when thickness was normalized for age and sex (*r* ^2 ^= 0.025, *p* = 0.330 and *r* ^2 ^= 0.064, *p* = 0.241, respectively). However, these two clinical measures of visual function were significantly correlated with each other (*r* ^2 ^= 0.333, *p* = 0.040).

**Table 3:** Bi-variate Pearson correlation analysis.

Table 3 Structure-Function Correlations	Latency (ms)	Acceleration (deg/s^2^)	Gain	Direction Noise (°)	Saccadic Rate (Hz)	Fixation Error (deg)	Mean Deviation	Pattern Standard Deviation
Raw Thickness	-0.595	0.473	0.483	-0.413	0.131	-0.448	0.0577	-0.1599
r^2^	35.4%	*22.3%*	*23.3%*	*17.1%*	1.7%	*20.1%*	0.3%	2.6%
p	0.0206	0.0603	0.0559	0.0910	0.3425	0.0720	0.4372	0.3295
Normalized Thickness	-0.3995	0.3969	0.2737	-0.3004	0.2300	-0.5055	0.1593	-0.2520
r^2^	*16.0%*	*15.8%*	7.5%	9.0%	5.3%	25.6%	2.5%	6.4%
p	0.0991	0.1007	0.1947	0.1713	0.2361	0.0468	0.3302	0.2412
Age	0.4634	0.0615	-0.4297	0.5270	-0.2844	0.0087	0.4942	-0.3861
r^2^	*21.5%*	0.4%	*18.5%*	27.8%	8.1%	0.008%	*24.4%*	14.9%
p	0.0646	0.4247	0.0816	0.0392	0.1852	0.4892	0.0733	0.1352
Mean Deviation	0.0080	0.0494	-0.6104	0.2084	-0.5052	-0.2935	1.000	-0.5775
r^2^	0.006%	0.2%	*37.3%*	4.3%	*25.5%*	8.6%	100%	33.3%
p	0.4913	0.4461	0.0609	0.2817	0.0682	0.2053	-	0.040224

This Table shows the Pearson’s correlation of 6 oculometrics, Mean Deviation (MD), and Pattern Standard Deviation (PSD) with Thickness, Normalized Thickness, Age, and Mean Deviation across the 24 retinae of our patient population (with df conservatively set to number of subjects minus 2, see Methods). Significant correlations (p < 0.05) have bolded r^2^ values and those that approach significance (p < 0.11) have italicized r^2^ values. Note that Latency, Acceleration, Gain, Direction Noise, and Fixation Error show a significant linear relationship with Thickness or Normalized Thickness (or one that approaches significance). Note that Latency, Gain, and Direction Noise show a significant linear relationship with Age (or one that approaches significance). Note that only Gain and Saccadic Rate show a linear relationship with MD that even approaches significance.

In contrast to MD and PSD, several oculometric measures of visuomotor function were at least somewhat correlated with Thickness (**Table 3**). In particular, Latency was significantly correlated with Thickness (*r* ^2 ^= 0.354, *p* = 0.021) but fell below significance when thickness was normalized (*r* ^2 ^= 0.160, *p* = 0.099). Conversely, Fixation Error was significantly correlated with Normalized Thickness (*r* ^2 ^= 0.256, *p* = 0.047) but fell below significance for raw Thickness (*r* ^2 ^= 0.201, *p* = 0.072). For Acceleration, the correlation with both Thickness and Normalized Thickness approached significance (*r* ^2 ^= 0.223, *p* = 0.060 and *r* ^2 ^= 0.158, *p* = 0.101, respectively). For Gain and Direction Noise the correlation approached significance for raw Thickness only (*r* ^2 ^= 0.233, *p* = 0.056 and *r* ^2 ^= 0.171, *p* = 0.091, respectively).

Two oculometric measures, Gain and Saccadic Rate, showed a correlation with MD that approached significance (*r* ^2 ^= 0.373, *p* = 0.061 and *r* ^2 ^= 0.255, *p* = 0.068, respectively), suggesting that they might capture aspects of visual function that overlap with MD. However, the remaining oculometric measures that either show impairment (**Table 2**) or correlation with Thickness (**Table 3**) showed little or no evidence of correlation with MD, indicating that they capture variation in visual function not captured by MD, at least at the early stage of toxicity.

Direction Noise was significantly correlated with Age (*r* ^2 ^= 0.278, *p* = 0.039) with Latency and Gain not quite so (*r* ^2 ^= 0.215, *p* = 0.065 and *r* ^2 ^= 0.185, *p* = 0.082, respectively). For MD, the correlation with Age also did not quite reach significance (*r* ^2 ^= 0.244, *p* = 0.073).

### Effects of thickness and age

**Figure 2:**
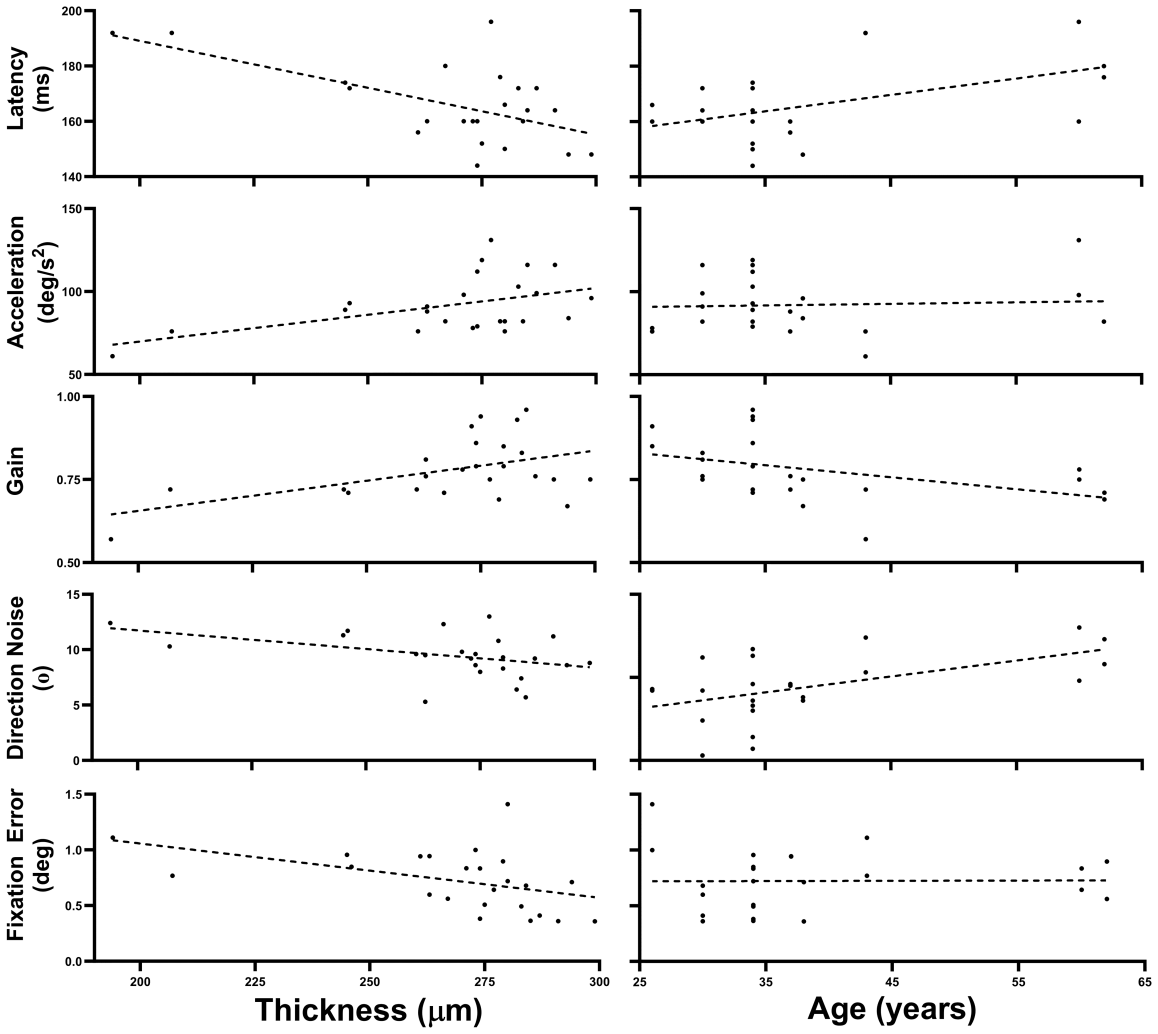
Thickness and Age trends. This figure plots the data across all 24 retinae for the five oculometrics from our patient population that showed a significant or nearly significant correlation with Thickness, plotted as a function of both Thickness and Age. The dashed lines are the best fitting linear-regression lines.

**Figure 2** illustrates the five oculometrics that demonstrated a significant or almost significant correlation with Thickness, as determined by our conservative bi-variate correlation analyses above. Given that Thickness is not correlated with Age (see **Figure 1** and 63), those metrics that correlate with both Age and Thickness likely have separate and largely independent relationships with these two factors. To conduct an analysis that is statistically more powerful than the correlation analysis above, and to segregate the magnitude of the Thickness and Age effects more cleanly, we used a Linear Mixed-Effect Model (LMM) analysis to quantify the effects of these two factors simultaneously. This multiple-regression analysis leverages the two measurements per subject, while also accounting for correlations across within-subject repeated measures from the two retinae, to yield maximum-likelihood estimates of the mean linear slopes of the two effects without exaggerating the degrees of freedom.

**Table 4** summarizes the effects of Thickness and Age across all 24 retinae tested for the 5 oculometric measures shown in **Figure 2**. Latency showed significant effects of Thickness (-0.301 ms/μm, *F(*1,13.15) = 11.109, *p* = 0.003) and Age (0.499 ms/year, *F(*1,11.91) = 5.979, *p* = 0.016). Acceleration showed a significant effect of Thickness (0.334 deg/s^2^/μm, *F(*1,12.969) = 6.898, *p* = 0.011), but not of Age (F(1,12.017) = 0.472, *p* = 0.253). Gain showed significant effects of Thickness (0.002/μm, *F(*1,14.569) = 5.02, *p* = 0.021) and of Age (-0.003/year, *F(*1,12.043) = 3.362, *p* = 0.046). For Direction Noise, the effect of Thickness did not quite reach significance (-0.025 °/μm, *F(*1,14.185) = 2.749, *p* = 0.060), but the effect of Age did (0.088 °/year, *F(*1,11.904) = 6.175, *p* = 0.015). Finally, Fixation Error showed a significant effect of Thickness (-0.005 deg/μm, *F(*1,14.485) = 3.758, *p* = 0.036), but not of Age (*F*(1,11.966) = 0.047, *p* = 0.416). Thus, aside from the correlation of Direction Noise with Thickness, which still did not quite reach significance, the LMM analysis showed that the effects of Thickness and Age that nearly reached significance using the simpler but conservative correlation analysis above, were indeed fully significant. For completeness, the LMM analysis also found a new hint of a linear trend with Thickness for Saccadic Dispersion (0.114 °/μm, *F(*1,16.862) = 2.913, *p* = 0.106), but no new significant effects were uncovered.

**Table 4:** Thickness and Age LMM slopes.

Table 4 - LMM slopes	Latency (ms)	Acceleration (deg/s^2^)	Gain	Direction Noise (°)	Fixation Error (deg)
Thickness (μm)	**-0.301**	**0.334**	**0.002**	-0.025	**-0.005**
Age (years)	**0.499**	N.S.	**-0.003**	**0.088**	N.S.

Entries in bold are statistically significant (p < 0.05) based on LMM analysis. The Thickness slope for Direction Noise is not quite significant (p = 0.06). We also used LMM to confirm that there was no direct relationship between Thickness and Age (F(1,12) = 0.236, p = 0.318).

## Discussion

### Visual and visuomotor deficits in asymptomatic SLE patients undergoing HCQ treatment

Our findings indicate significant disruption in various aspects of visuomotor behavior in a cohort of 12 asymptomatic SLE patients. In particular, the initial acceleration of the pursuit response, a measure of open-loop gain, which reflects the vigor of the initial neural response without the benefit of closed-loop visual feedback (25), is reduced by 22%. Furthermore, despite visual feedback driving tracking corrections, steady-state pursuit gain remains impaired, showing a 16% reduction. This is consistent with the observed large, presumably compensatory, 46% increase in catch-up saccade amplitude associated with a 7% decrease in proportion smooth. Speed responsiveness was also decreased by 31%, indicating that the processing of target motion speed is impaired. Lastly, an observed 46% increase in fixation error and a hint of an increase in saccadic dispersion provide evidence of decreased static and dynamic parafoveal spatial localization, respectively.

Our correlation and LMM analyses show that several oculometrics have a significant linear relationship to retinal thickness in our patient population (Latency, Acceleration, Gain, and Fixation Error, with Direction Noise nearly reaching significance). Of those metrics showing deficits in our patient group and/or correlation with Thickness, only Gain shows any indication of correlation with MD, suggesting that the remaining oculometrics are capturing effects on visual function (e.g., motion processing, spatial localization) that are not captured by MD (a measure of light sensitivity) and thus represent new and independent information about variation in visual function.

### Visual pathologies associated with SLE and with HCQ treatment

SLE patients can exhibit disruptions in visuomotor behaviors due to auto-immune pathology in both the afferent and efferent limbs of their ocular tracking response. While anterior segment disease like dry eye or conjunctivitis is common and generally not debilitating, it can serve as an early indicator of disease activity (65–67). These manifestations are primarily inflammatory in nature and can range from mild to severe, although they are generally not expected to cause significant visual disability unless they become severe (68). The posterior segment of the eye presents a more nuanced picture, with involvement potentially including more severe complications such as retinal vascular changes, optic neuritis, and occlusive vasculitis, which can lead to visual field defects and central vision loss (65–69). Lastly, oculomotor efferent commands can also be altered by pathology of the oculomotor muscles and surrounding orbital tissues (70) or of the neural processing of pre-motor signals (71–73). Thus, there are a number of potential physiological mechanisms by which SLE itself could cause compromise of visuomotor responses similar to those we observed. However, ophthalmic examination of our patient cohort as well as acuity testing cleared them of any significant compromise of the anterior eye. Similarly, the absence of any observed deficit of pupillary light responses as well as normal optic nerve examination suggests that there is no gross compromise of the optic nerve. The absence of any observed deficit of eccentric gaze holding as well as the healthy neurological status of our asymptomatic patient cohort suggest there is little compromise of oculomotor efferent pathways. Finally, previous human psychophysical studies (28, 30, 32) have shown a direct quantitative relationship between variation in oculometrics and visual perception showing that oculomotor performance is typically limited by visual processing sensory input noise, not motor output noise (74, see however, 31). We conclude that the observed oculometric deficits in our patient cohort are most likely due to impairment of visual processing, either in the retina or central visual processing pathways or both, but a subtle motor contribution cannot be ruled out.

Anti-malarial drugs, such as HCQ, represent a standard first-line treatment for SLE because they have been shown to be very effective in relieving symptoms and slowing the progression of disease (75). However, HCQ treatment is independently associated with retinal toxicity, with its initial signature effects on parafoveal vision that start in the outer layers of the retina, progress to the Retinal Pigment Epithelium, and can ultimately lead to serious impairment of foveal vision with continued exposure (76). The standard clinical practice is to surveil these patients with testing that includes automated perimetry and spectral domain OCT, with particular attention to the parafoveal outer retina. However, given that more advanced stages of toxicity can progress even after treatment cessation (77), it is imperative to identify toxicity early and, if possible, to distinguish it from preexisting or concurrent retinal pathologies, either due to SLE directly or other comorbidities, independent of HCQ treatment. Although our data show functional visual deficits even in the absence of overt structural signs of HCQ toxicity, it is possible that some or all of our findings are due to subtler toxicity effects that precede structural damage that can be seen in OCT.

### Potential causes of the observed visuomotor deficits in our patient group

While these results demonstrate clear deficits in visuomotor performance in our patient group, we cannot be sure of the cause(s) of these deficits, as they could either be due to SLE itself, the HCQ treatment, both, or potentially even something else about our patient cohort that is systematically different from our cohort of healthy age-matched controls. However, the fact that Fixation Error is 7% smaller and Gain 4% larger when the two patients with identified clinical evidence of toxicity are excluded, suggests that the parafoveal static localization and dynamic stabilization functions associated with these two metrics, respectively, may be more specifically associated with HCQ toxicity than to SLE, consistent with toxicity being related to photoreceptor pathology especially in the parafoveal region. A similar argument could be made for Saccadic Amplitude.

The observed correlation with Thickness suggests that the observed deficits in Acceleration, Gain, and Fixation Error are likely due to disruption of neural processing within the retina. However, it does not resolve the issue of whether this is due to SLE directly or to HCQ treatment. Conversely, the absence of correlation of Speed Responsiveness with Thickness suggests that its observed impairment may be due to non-retinal, central effects, and thus to SLE directly, consistent with the common view that speed estimation is a cortical phenomenon (78) and that HCQ toxicity is not known to have central effects. Finally, the observed increase in Saccadic Amplitude (and decrease in Proportion Smooth) is not likely due to a neural deficit at all, but rather likely represents a healthy compensatory saccadic response to the observed deficit in pursuit acceleration and gain.

Our negative findings indicate that many visual pathways appear spared by both SLE and HCQ treatment in our patient group. In particular, direction processing appears normal so, given the normal pursuit latency, it would seem that the earliest portion of the directionally selective responses emerging from the retina is healthy. Similarly, given the healthy pupillary light responses, there is no evidence of significant damage to the subcortical visual pathways driving that response (79), including the melanopsin-containing ganglion cells that transduce light for the regulation of circadian rhythms (80), although more extensive pupillary response testing (using longer and chromatic stimuli) would be necessary to carefully dissect the health of these various pathways. While it is impossible from oculometrics alone to pinpoint the neural locus(ci) of the compromise in initial acceleration, gain, and speed responsiveness of pursuit, the locus for impaired acceleration must be relatively early in the visual pathway, no later than early extrastriate cortex such as the Middle Temporal area (81), while that for the latter two could be later, perhaps in more rostral extrastriate cortex (82) or even in frontal cortex (83). However, because retinal signals feed all of these pathways, our data cannot rule out the possibility that all of the observed deficits in this study are entirely due to the disruption of neural signals within the retina without additional cortical involvement. That said, the healthy direction processing evident in **Table 2** supports the possibility that the altered speed response may be due to central effects associated directly with SLE.

### Caveats

Our cohort size is small, making generalization to the population-at-large somewhat tenuous. That said, many of the effects reported here were large and robust, representing a solid basis to motivate a larger study. The small sample size also may have caused us to miss subtler findings, so the effects that did not quite reach significance reported above as well as others may be revealed as significant in future larger studies. Furthermore, our behavioral paradigm only tested a limited range of retinal eccentricities so it may have missed retinal or other visual-field pathologies beyond the parafovea. Although the findings above suggest that the pattern of impaired vs. spared function across our set of oculometrics may shed light on the locus and origin of subtle visuomotor impairment to potentially assist in diagnostic specificity, because we did not have a control group with SLE but not taking HCQ, this study cannot conclusively determine the extent to which our various observations were due to effects of HCQ toxicity or of SLE directly. We also cannot entirely rule out the possibility that the performance of our limited control group was skewed by happenstance toward better performance thus biasing our impairment findings. However, the well-matched values between our patient and control cohorts for the many unaffected oculometrics in **Table 2** and the fact that performance of our patient group remains inferior for the 5 affected oculometrics tested previously (i.e., all but Fixation Error) when compared to an unmatched set of baseline data from 43 normal subjects pooled across prior recent studies (58) argue against this possibility, with only Gain showing a hint that this might be the case. Furthermore, any potential skew in our control data would not impact our within-subject correlation findings. Further studies with larger control groups and patient populations with known pronounced SLE symptoms (or who have never received HCQ treatment) and/or who are experiencing more advanced HCQ toxicity, where there is independent confirmation of disease and/or drug-mediated mechanisms, will be necessary to resolve conclusively the important follow-up question of whether the observed deficits are due to SLE or HCQ.

We must also emphasize that we are not implying that the 10 patients without diagnosed toxicity showed no clinical deviations from normal using standard clinical measures. Indeed, marginal thinning, reduced contrast sensitivity, or a somewhat abnormal ERG (in one of 4 patients tested) were found for three patients in this subgroup. However, none of these 10 patients were diagnosed to have toxicity based on the constellation of standard clinical measures, and thus were not recommended to discontinue HCQ. It would clearly be valuable for a future study to correlate ERG and oculometric measures in populations at-risk for retinal pathology.

It must also be noted that our patient group was entirely female (randomly due to a large sex bias in the prevalence of SLE), while our healthy control group was largely balanced male and female. A preliminary meta-analysis of the combined baseline data from several previous studies examining the same set of oculometrics under binocular viewing conditions found only subtle differences in performance based on sex in a healthy population of 43 subjects, and only one small but significant main effect of sex: increased Saccadic Dispersion for females (58). A preliminary assessment of the effect of sex on the oculometrics of our control cohort of 17 subjects under monocular viewing conditions only found a small but significantly lower Saccadic Rate for females. Although it is possible that our use of a mixed-sex control group obscured the findings for Saccadic Rate or Saccadic Dispersion, when our control baseline is restricted to include only the 8 females, neither Saccadic Dispersion nor Saccadic Rate show significant differences from the patient group, consistent with the data in **Table 2**. These preliminary observations of potential secondary effects of sex are far from definitive and the resolution of potential sex confounds for some oculometrics awaits a balanced-sex study on a larger population of patients that includes male SLE patients. That said, it should be noted that our prior data provide no indication of a sex effect for the 6 oculometrics significantly impaired in our patients (**Table 2**) or for the 5 oculometrics showing meaningful correlations with Thickness (**Figure 2** and **Table 4**).

Lastly, our correlation and LMM analyses were exclusively on the patient population so we cannot determine to what extent these correlations were due to shared normal versus pathological variance. Our conclusions about correlations are therefore restricted to demonstrating that 17–35% of the total variance in Thickness in our patient group is shared with that of the oculometric measures in **Figure 2** and **Table 4**, indicating that they are capturing some of the same physiological variance, thereby linking the structural and functional findings in our patient population.

## Conclusions

The fact that four of our oculometric measures show significant and considerable (12 to 41%) impairment in a cohort of asymptomatic patients without diagnosed toxicity, demonstrates that oculometrics have greater sensitivity for the early detection of visual deficits than current ophthalmological surveillance methods. This is not surprising as structural changes visible in OCT require either cell death or other gross changes in cellular morphology, while oculometrics can detect small changes in synaptic efficacy that may be devoid of structural manifestations or be associated with structural changes that are only visible at the electron microscopy level. Furthermore, the fact that all but one of the six altered oculometrics in our patient population do not show even a hint of correlation with MD, the current gold-standard clinical tool for visual-function testing, suggests that these oculometrics provide different and relevant information about visual function not currently available to ophthalmologists.

Many of the observed functional deficits in our patient population are consistent with retinal toxicity due to the shared parafoveal nature of the expected toxicity and of the affected oculometrics and because many of the impaired oculometrics show significant correlation with retinal thickness. If these deficits are indeed due to toxicity, then our oculometric testing is detecting HCQ toxicity with greater sensitivity than OCT or perimetry. However, even in the unlikely case that the observed deficits are entirely due to the underlying SLE itself, our oculometric testing has instead uncovered significant visual/visuomotor impairment in a random sample of asymptomatic SLE patients (see 84, 85). Regardless of the cause or locus of the deficits, we conclude that oculometrics represents a novel set of biomarkers of retinal and visual health that could form the basis of promising new clinical tools, with high sensitivity as well as potential specificity, to assist ophthalmologists in the detection and interpretation of retinal compromise when surveilling at-risk patients, such as those with SLE on long-term HCQ treatment, as well as neurologists and rheumatologists concerned about potential subtle central visuomotor effects.

## Data availability statement

Anonymized data will be provided by request to the extent authorized by NASA and its human research institutional review board. Requests to access the datasets should be directed to the corresponding author at Leland.S.Stone@nasa.gov.

## Ethics statement

The Stanford portion of the human research data collection was conducted at the Byers Eye Institute, Stanford University School of Medicine, and was approved by the Institutional Review Board of Stanford University. The NASA portion of the human research data collection was conducted at the National Aeronautics and Space Administration, Johnson Spaceflight Center, and was approved by the NASA Human Research Institutional Review Board. The studies were conducted in accordance with the local legislation and institutional requirements. The participants provided their written informed consent to participate in this study.

## Author contributions

AB: Data curation, Formal analysis, Visualization, Writing – original draft, Writing – review & editing. KS: Writing – original draft, Writing – review & editing, Data curation, Formal analysis, Funding acquisition. MA: Writing – review & editing, Software, Validation. RG: Conceptualization, Writing – review & editing. TT: Writing – review & editing, Software. YA: Writing – review & editing, Supervision. MA: Writing – review & editing, Supervision. LL: Supervision, Writing – review & editing, Conceptualization, Funding acquisition, Methodology, Resources, Formal analysis. LS: Supervision, Writing – review & editing, Conceptualization, Formal analysis, Funding acquisition, Methodology, Resources, Writing – original draft.
